# Intersecting Mechanisms of Hypoxia and Prostaglandin E2-Mediated Inflammation in the Comparative Biology of Oral Squamous Cell Carcinoma

**DOI:** 10.3389/fonc.2021.539361

**Published:** 2021-05-21

**Authors:** Walaa Hamed Shaker Nasry, Chelsea K. Martin

**Affiliations:** Department of Pathology and Microbiology, Atlantic Veterinary College, University of Prince Edward Island, Charlottetown, PEI, Canada

**Keywords:** OSCC (oral squamous cell carcinoma), hypoxia, inflammation, comparative oncology, treatment resistance, arachidonic acid pathway

## Abstract

The importance of inflammation in the pathogenesis of cancer was first proposed by Rudolph Virchow over 150 years ago, and our understanding of its significance has grown over decades of biomedical research. The arachidonic acid pathway of inflammation, including cyclooxygenase (COX) enzymes, PGE2 synthase enzymes, prostaglandin E2 (PGE2) and PGE2 receptors has been extensively studied and has been associated with different diseases and different types of cancers, including oral squamous cell carcinoma (OSCC). In addition to inflammation in the tumour microenvironment, low oxygen levels (hypoxia) within tumours have also been shown to contribute to tumour progression. Understandably, most of our OSCC knowledge comes from study of this aggressive cancer in human patients and in experimental rodent models. However, domestic animals develop OSCC spontaneously and this is an important, and difficult to treat, form of cancer in veterinary medicine. The primary goal of this review article is to explore the available evidence regarding interaction between hypoxia and the arachidonic acid pathway of inflammation during malignant behaviour of OSCC. Overlapping mechanisms in hypoxia and inflammation can contribute to tumour growth, angiogenesis, and, importantly, resistance to therapy. The benefits and controversies of anti-inflammatory and anti-angiogenic therapies for human and animal OSCC patients will be discussed, including conventional pharmaceutical agents as well as natural products.

## Introduction

Hypoxia, defined as reduced oxygen levels or deficiencies in oxygen transport, is a common feature of solid tumours, and plays an important role in triggering the development of new blood vessels (angiogenesis), which is critical for supporting tumour growth. Importantly, hypoxia and production of reactive oxygen species (ROS) can serve as drivers of inflammation (1). Additionally, tumour associated inflammation can lead to activation of hypoxia-inducible factors and pathways that help support tumour progression (2). One of the key mechanisms of tumour-associated inflammation is activity of the arachidonic acid pathway resulting in generation of prostaglandin E_2_ (PGE2). Increased PGE2 secretion is usually attributed to increased expression and activity of cyclooxygenases 1 and 2 (COX-1 and COX-2), but there are a number of other points in the arachidonic acid pathway that can influence PGE2 production and there are a variety of PGE2 receptors that are responsible for PGE2-related responses (3). Areas of chronic hypoxia have been identified in the tumours of patients with oral cancer (4). Targeting the cycle of inflammation and hypoxia may represent an opportunity to slow tumour progression and improve therapeutic outcomes for patients diagnosed with oral and oropharyngeal squamous cell carcinoma (OSCC).

OSCC is the 6th most common form of cancer worldwide (5), and has an overall 5-year survival of only 63% (6). Despite decades of research, improved therapies are desperately needed for patients battling this aggressive form of cancer. We hypothesise that the arachidonic acid pathway of inflammation and hypoxia possess overlapping activities and mechanisms that might serve as ideal therapeutic targets. The presence of overlapping mechanisms also raises the possibility that certain therapeutic strategies may be rendered less effective under certain conditions (such as in areas of tumour hypoxia). The purpose of this review is to explore the scientific literature related to the interaction of hypoxia and the arachidonic acid pathway of inflammation, in order to improve our understanding of how these interactions contribute to OSCC disease progression and treatment resistance.

**Figure 1:**
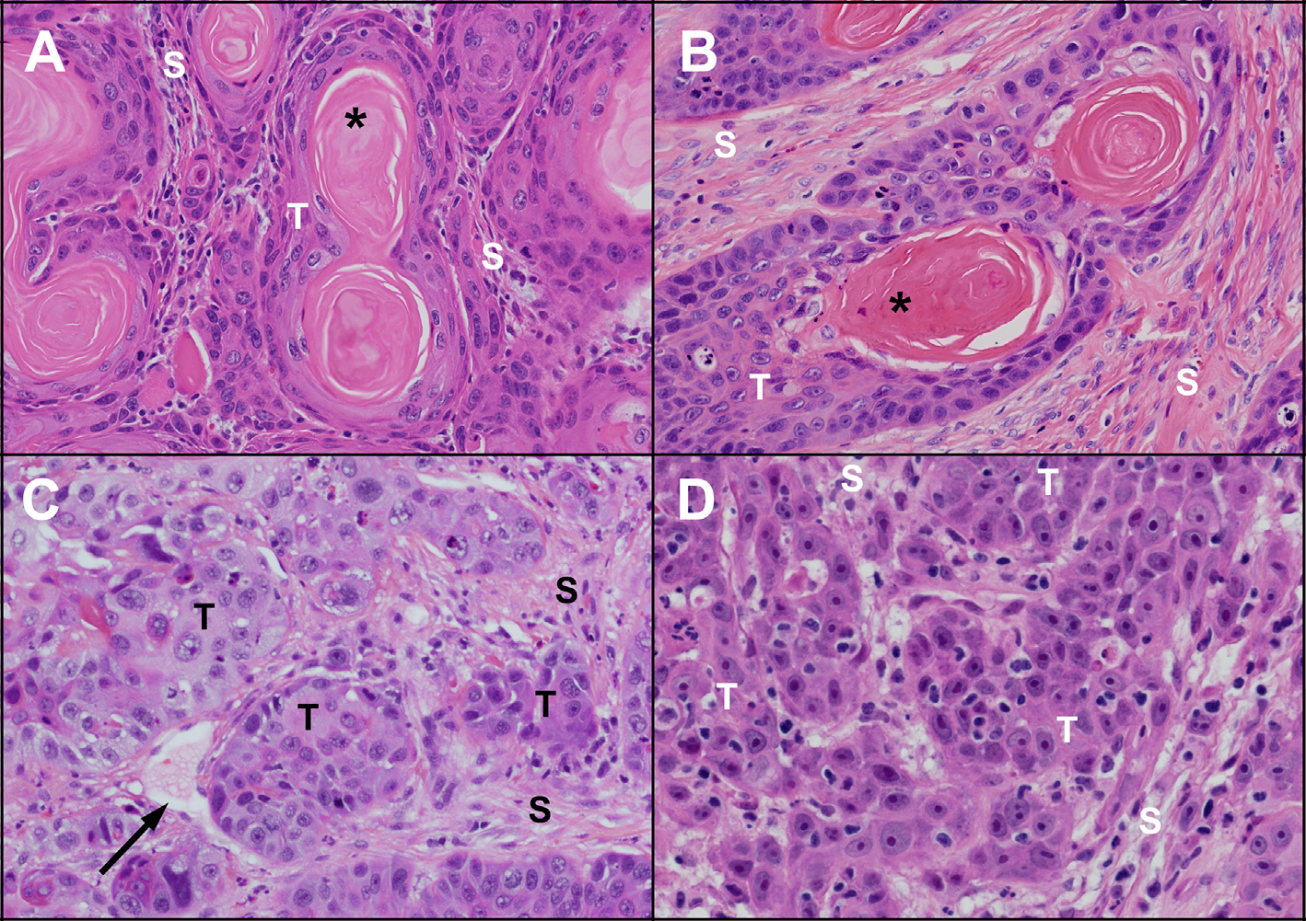
Microscopic appearance of untreated human and feline OSCC. Human **(A)** and feline **(B)** OSCC tumours (T) with clusters of well differentiated malignant squamous epithelial cells showing progression from peripheral basal-like cells through stratified squamous epithelium with keratinization forming a central ‘keratin pearl’ (*). Islands of OSCC cells are surrounded by supporting stroma (S) composed of fibrous connective tissue (fibroblasts and collagen). Less differentiated human **(C)** and feline **(D)** OSCC are composed of irregular islands and trabeculae of tumour cells (T) lacking orderly arrangement and keratin pearls. A thin walled vessel (vein or lymphatic) is indicated by the black arrow. (haematoxylin and eosin stain, 40X objective).

Development of OSCC is not limited to humans. In fact, OSCC is diagnosed in domestic animals and is of particular significance in feline oncology. Feline OSCC (FOSCC) is the most common form of cancer in the oral cavity of cats, and is similar to human OSCC in several ways including microscopic appearance (**Figure 1**), invasiveness, poor prognosis, and expression of mediators including cyclooxygenase (COX) enzymes and CD147 (involved in inflammation and invasion) (7, 8). Research resulting in improved outcomes for pet cats with FOSCC, has the potential to improve outcomes for human OSCC patients as well.

A natural model of OSCC in cats can improve human OSCC research by complimenting existing experimental animal models. For example, immunocompromised rodent models are extensively used in human cancer research, but the animals do not have the genetic diversity and intact immune systems that human cancer patients have. Therefore, this literature review includes a discussion of the comparative biology of these mechanisms in people and in veterinary patients, especially in cats.

**Figure 2:**
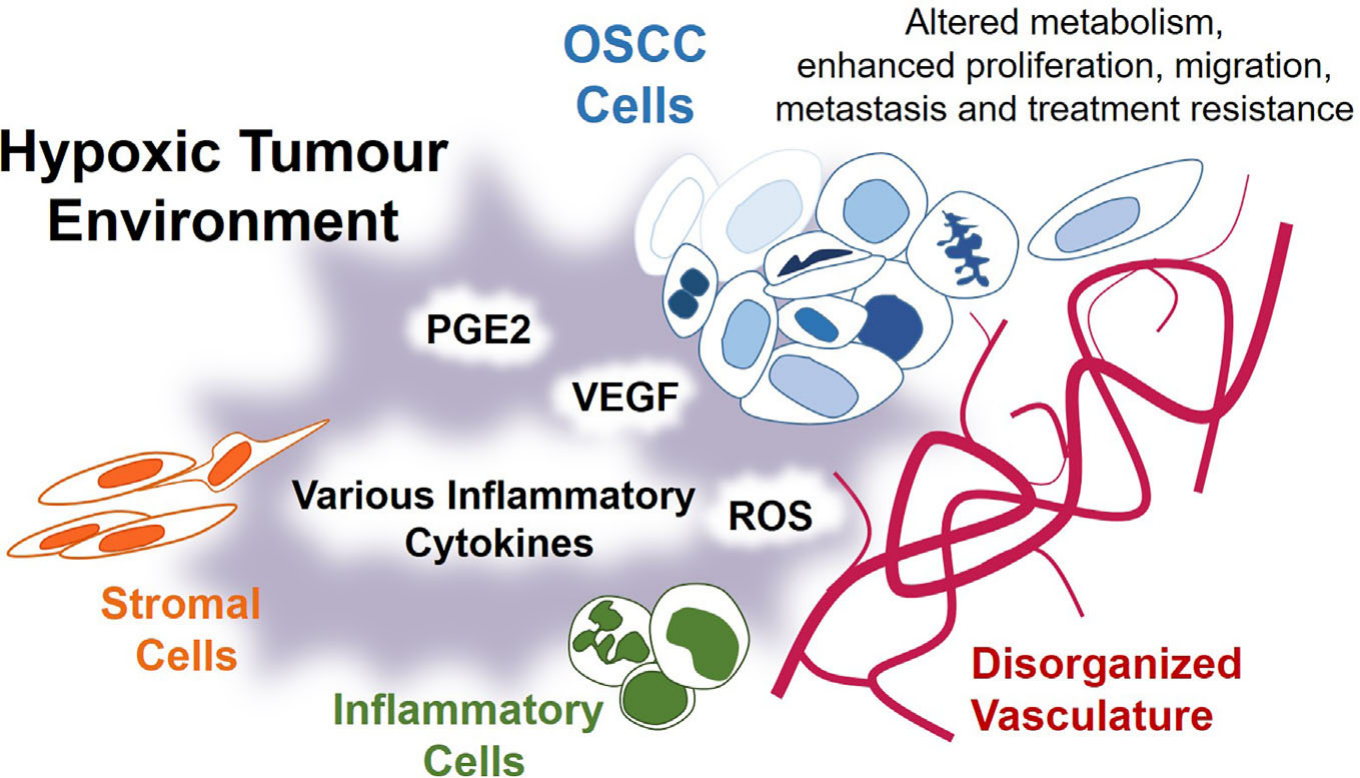
Introduction to hypoxia and inflammation in OSCC. OSCC tumours are composed of a variety of cell types, which include vascular endothelial cells, inflammatory and immune cells, stromal cells, and OSCC tumour cells. This literature review explores the relationship between hypoxia and inflammation in OSCC tumours, as well as how this relationship contributes to disease progression. Key products of this relationship include inflammatory PGE2 (prostaglandin E2), angiogenic VEGF (vascular and endothelial growth factor), ROS (reactive oxygen species). Throughout this literature review, mechanisms leading to the synthesis of these factors will be described, with emphasis on how these mechanisms interact and overlap. An understanding of these interactions is important to the process of developing new therapeutic strategies for OSCC in humans and in animals.

We recently reviewed the roles of PGE2 in development and progression of OSCC (3) and readers are referred to other publications for recent literature dedicated to the general role of hypoxia in OSCC (9, 10). Here, we focus on the overlapping and intertwined mechanisms of inflammation and hypoxia in OSCC that contributes to disease progression and treatment resistance (**Figure 2**).

Despite efforts of the scientific community to find chemoprotective or chemotherapeutic strategies for OSCC, including conventional pharmaceuticals and naturally-derived products, there is still controversy about the safety, bioavailability, and effectiveness of these therapies. These therapeutic considerations will also be covered in this comparative oncology review. We are emphasizing OSCC-specific literature, but references to other forms of cancer are included when they include relevant findings that have not yet been reported in OSCC.

## Cancer-Related Roles of Hypoxia and Angiogenesis in OSCC

### Overview of Tumour Hypoxia

**Figure 3:**
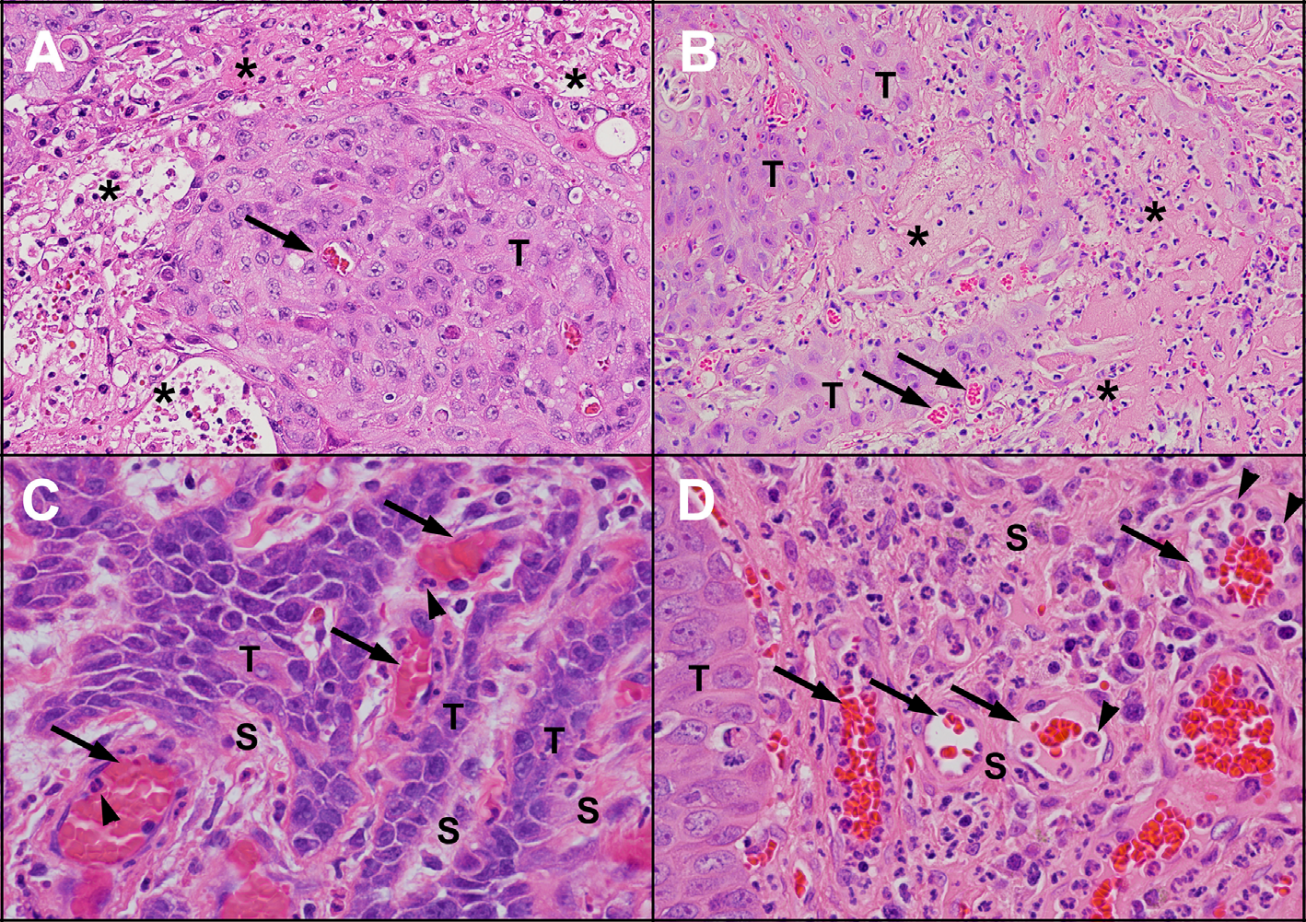
Areas of necrosis and new blood vessel formation in untreated human and feline OSCC. Human **(A)** and feline **(B)** OSCC tumours showing transition from intact, viable-appearing OSCC tumour cells (T) to areas of necrosis (*) characterised by loss of structure and replacement with eosinophilic (pink) proteinaceous material and cellular debris. Small blood vessels are present near the tumour cells (arrows). One of the causes of tumour necrosis is hypoxia. Within the stroma (S) of human **(C)** and feline **(D)** OSCC, numerous blood vessels are present (arrows), indicating growth of new blood vessels into the region. In both **(C, D)**, inflammatory cells (neutrophils, arrowheads) are marginating along the endothelial surface. [haematoxylin and eosin stain, 20X objective **(A, B)** and 40X objective **(C, D)**].

In tumours, disturbed microcirculation due to structural abnormalities of a tumour vascular network is an essential contributor to hypoxia, as reviewed previously (11). In the tumour microenvironment, there are two types of hypoxia; chronic hypoxia and cyclic hypoxia. Chronic hypoxia was first described in 1955, when Thomlinson and Gray noticed the presence of necrotic areas surrounded by intact tumour cells, corresponding to a falling gradient in oxygen tension between the periphery and the centre of tumours (12). Similar features can be found in OSCC (**Figure 3**). Chronic hypoxia occurs when tumour cell proliferation exceeds what the blood vascular network can support (11). The second type of hypoxia is cyclic hypoxia, which is caused by fluctuations in tumour perfusion, and was first described by Brown In 1979 (13). Repeated fluctuations between periods of hypoxia and reoxygenation trigger production of reactive oxygen species (ROS). Cyclic hypoxia can be caused by high-frequency hypoxia cycles or low-frequency hypoxia cycles. High-frequency cycles are due to transient fluctuations in perfusion, and low-frequency hypoxic cycles are caused by vascular remodelling (dynamic changes occurring in the microvascular structure) within a tumour vascular network on a daily basis (14). A study in OSCC patients showed that areas of acute and chronic hypoxia within a tumour can change over time, and may contribute to radiation therapy resistance (4). Contributions of hypoxia to treatment resistance is discussed in more detail in section 4.2.

Cyclic hypoxia is particularly significant because it conditions endothelial cells to become more resistant to stress compared to endothelial cells in chronic or normoxic conditions. Additionally, cyclic hypoxia is associated with a greater upregulation of hypoxia-inducible factor 1 (HIF-1), a transcription factor important in the development of new blood vessels, compared to chronic hypoxia (15). Furthermore, production of damaging ROS is stimulated by cyclic hypoxia to a much greater extent than chronic hypoxia (16). Interestingly, hypoxia is not the only condition within tumours that can contribute to the accumulation of ROS; tumour-associated inflammation is also known to stimulate ROS production.

### Hypoxia Inducible Transcription Factors

The HIF family of transcription factors has three distinct members, HIF-1, HIF-2, and HIF-3. They all contain an oxygen-sensitive HIFα subunit (HIF-1α, HIF-2α or HIF-3α), which dimerises with the constitutively expressed HIF-1β subunit. In the presence of oxygen, HIFα subunits become hydroxylated, which causes them to bind to von Hippel-Lindau tumour suppressor (VHL) and ultimately leads their degradation. Similar to HIF-1α and HIF-2α, HIF-3α levels are increased during periods of hypoxia, however HIF-3α appears to antagonise the function of HIF-1α and HIF-2α (17).

VHL is the causal gene of von Hippel-Lindau disease, which is a hereditary neoplastic syndrome characterised by susceptibility to hyper-vascular tumour development in multiple organs such brain, pancreas, adrenal gland, and kidney, as well as clear-cell renal cell carcinoma. The gene mutation is either a germline mutation which is responsible for disease inheritance, or a sporadic mutation (18, 19). Sporadic mutation of VHL has been shown in a study of patients with OSCC of the tongue (20), and another OSCC study showed that HIF-1α expression was inversely related to VHL expression and was positively associated with invasion, metastasis and poor prognosis (21). Further evidence for the importance of HIF-1α in the pathogenesis of OSCC comes from Chen et al. (22) who showed that that HIF-1α polymorphisms are associated with increased susceptibility to OSCC (22).

Although most studies indicate a positive relationship between HIF-1α and poor prognosis in people with OSCC, conflicting evidence exists. For example, in patients surgically treated for OSCC, high levels of HIF-1α expression appeared to have a beneficial effect of improved survival, but this was not observed for HIF-2α expression (23). In contrast, a different study revealed that OSCC patients with high HIF-2α had better outcomes than patients with low HIF-2α expression (24).

HIF transcription factors are not only regulated by hypoxia, they can also be upregulated by inflammation. For example, Yoshimura et al. (25) found that HIF-2α was related to COX-2 expression in colorectal cancer, which was in turn related to tumour aggressiveness (25). In a lung cancer cell line, PGE2 induced HIF-1α and expression of proangiogenic vascular and endothelial growth factor (VEGF) (26). Interestingly, hypoxic conditions increased the expression and activity of HIF-1 in OSCC cells *via* nuclear factor kappa-light-chain-enhancer of activated B cells (NFκB), leading to additional HIF-1 expression as well as expression of inflammatory cytokines such as interleukin (IL)-6 and IL-8 (27), which have been shown to be indicators of OSCC prognosis (28). Within the tumour, OSCC cells are not the only potential source of inflammatory cytokines; studies in other forms of cancer have shown that tumour associated inflammatory cells also produce cytokines, which can activate transcription factors such as NFκB that activate genes controlling cell survival, proliferation, growth, angiogenesis, invasiveness, and motility (29–31). Details of the intersecting mechanisms linking hypoxia and inflammation are discussed in section 3. The result of HIF transcription factor activation is increased expression of the VEGF family of growth factors, leading to the formation of new blood vessels in a process called angiogenesis (32, 33).

### Vascular and Endothelial Growth Factors (VEGFs) and Angiogenesis

Angiogenesis is the formation of new blood vessels, typically occurring during embryonic development, organ homeostasis, and in certain diseases (34). Normally, angiogenesis is a highly ordered process under tight regulation, relying on a balance between pro-angiogenic factors such as VEGF, fibroblast growth factors (FGFs), transforming growth factor-beta (TGF-β), and angiopoietin-1 and 2; and anti-angiogenetic factors such as angiostatin and platelet factor 4) (35, 36). In cancer, this balance becomes disturbed, and the resulting vessels are dilated, convoluted, and are highly permeable due to lack of functional pericytes, enlarged endothelial gaps, and an incomplete basement membrane (37, 38). **Figure 3** provides examples of prominent vascularity in OSCC.

Expression of VEGF in OSCC tumours is correlated with prognosis, lymph node metastasis, clinical stage, and low survival (39). Meta-analysis of twelve studies showed that VEGF positivity correlated with worse overall survival in OSCC patients, with the risk of death at 24 months being 1.88-fold higher in the patients with VEGF-positive tumours (40). It seems logical that increased VEGF and vascularisation of tumours would contribute to poor prognosis, yet VEGF appears to be capable of supporting OSCC progression in other ways. For example, VEGF has been shown to stimulate phosphoinositide 3′ kinase (PI3K)/protein kinase B (PKB/Akt) intracellular signalling leading to enhanced migration of OSCC cells (41).

The VEGF family consists of five secreted proteins including VEGF-A (usually referred to simply as VEGF), VEGF-B, VEGF-C, VEGF-D and placental growth factor (PLGF) (42–44). VEGF members bind tyrosine kinase receptors referred to as VEGFR1 (Fms-like tyrosine kinase 1; Flt1), VEGFR2 (foetal liver kinase 1; Flk1) and VEGFR3 (Fms-like tyrosine kinase 4; Flt4). Ligand binding of these receptors activates a complex network of intracellular signal transduction pathways such as extracellular signal-regulated kinase (ERK)/mitogen-activated protein kinase (MAPK), PI3K/Akt, and the stress kinase p38 MAPK (45). These signalling pathways go on to trigger cellular responses such as proliferation, migration, survival, and vascular permeability (46).

VEGF family members have different binding affinities for the VEGF receptors (47). VEGF-A, VEGF-B and PLGF bind to VEGFR1, VEGF-A also binds to VEGFR2, and VEGF-C and -D bind to VEGFR3. Vascular endothelial cells express VEGFR1, VEGFR2 and VEGFR3, however lymphatic endothelial cells also express VEGFR3 (48, 49). VEGFR1 is interesting in that it exists as a transmembrane isoform and as a soluble isoform. Binding of either VEGFR1 isoform with VEGF-A can influence the amount of VEGF-A available to bind VEGFR-2 or VEGFR-3, and therefore it can act as a negative or positive regulator of VEGF signaling (50).

Peterle et al. (51) revealed that VEGF-A is a prognostic tumour marker in OSCC patients (51). Elevations in VEGF-A, along with the inflammatory cytokine IL-6, in OSCC tumours has been attributed to increased IL-17, which was correlated with disease progression and a reduced overall survival (52). Interestingly, invasive and osteolytic (bone destructive) behaviour of OSCC cell lines was increased by VEGFR1 signaling (53). When considering the interaction between angiogenesis and inflammation, it is important to note that the levels of VEGF-C and -D can be upregulated by PGE2 within the tumour microenvironment (54). Other angiogenic factors, such as angiogenin (ANG), can also contribute to hypoxia-induced neovascularisation (55). ANG was increased by hypoxia in OSCC cell lines and was correlated with increased HIF-1 expression, demonstrating the potential importance of ANG in OSCC-associated angiogenesis (49).

In summary, tumour growth causes increased cellular demand for nutrients and oxygen, leading to hypoxia and HIF activation, which in turn stimulates angiogenesis *via* VEGF family members and their corresponding receptors. This type of angiogenesis is if often disorganized in tumours, leading to continued cycles of hypoxia and HIF activation (56–58). Treating OSCC with the help of antiangiogenic therapy will be discussed section 4.

## Interaction of Hypoxia and Inflammation in OSCC

### Overview of Hypoxia and Inflammation Crosstalk

**Figure 4:**
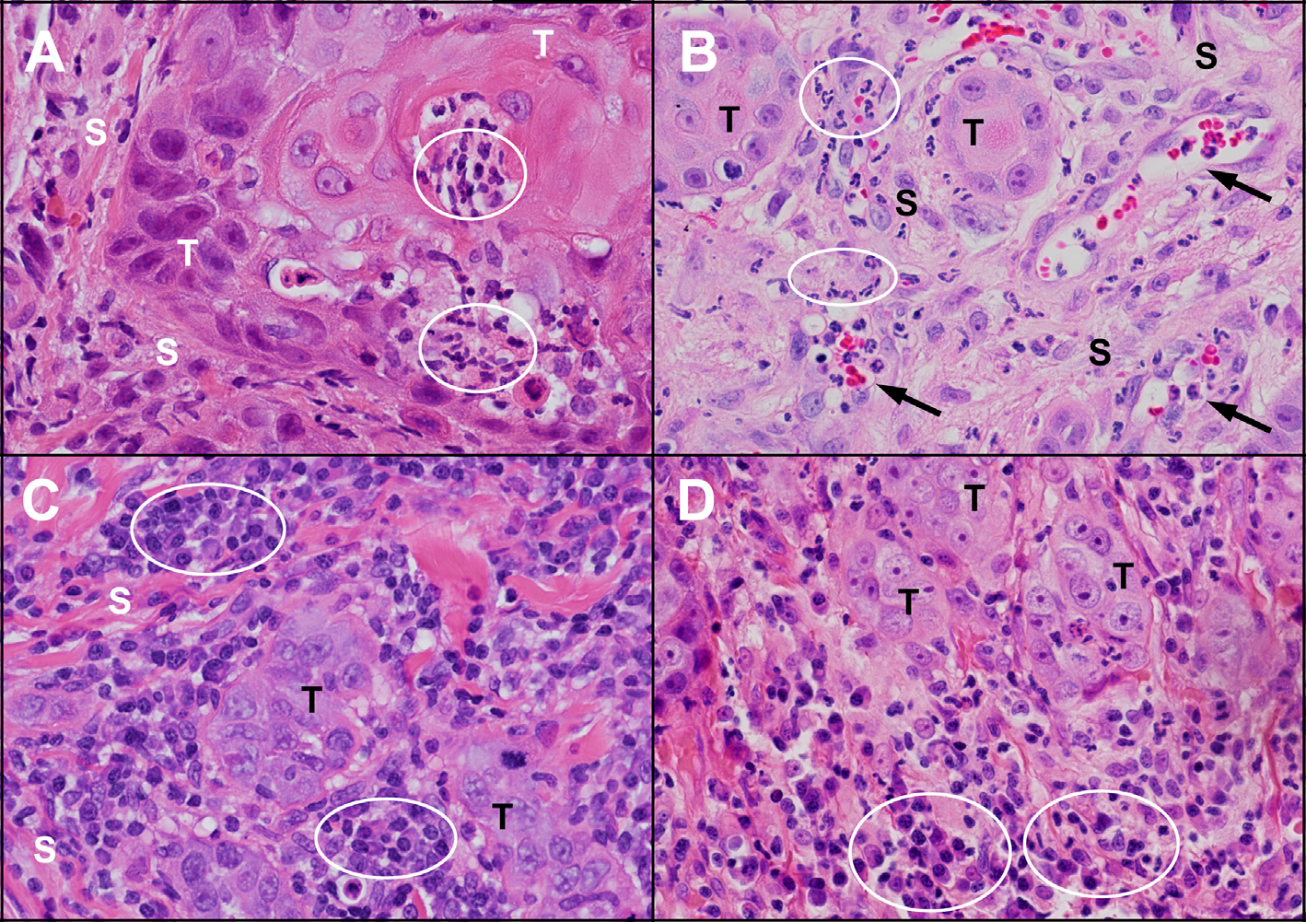
Inflammation in untreated human and feline OSCC. **(A)** An island of human OSCC tumour cells (T) embedded in stroma (S) with infiltrating neutrophils (circles). **(B)** Islands of feline OSCC tumour (T) with neutrophils (circles) infiltrating the tumour stroma (S) and within small blood vessels (arrows). **(C)** Small islands of human OSCC tumour cells (T) surrounded by numerous lymphocytes and plasma cells (circles). **(D)** Small islands of feline OSCC tumour cells (T) associated with mixed inflammatory infiltrates composed of plasma cells, lymphocytes and neutrophils (circles). (haematoxylin and eosin stain, 40X objective).

Inflammation in OSCC tumours is characterised on a cellular level by infiltration with inflammatory cells including neutrophils, lymphocytes and plasma cells (**Figure 4**). Hypoxia and inflammation work together in tumorigenesis, with the survival of cancer cells dependent on the regulation of the gene expression that controls antioxidant mechanisms in the tumour microenvironment (59, 60). Generally speaking, hypoxia and associated ROS generation can lead inflammation *via* a variety of mediators and pathways, such as tumour necrosis factor (TNF), interleukin-6 (IL-6), and inflammasome activation (1). One of the key ways that tumour hypoxia leads to inflammation is through activation of the transcription factor, nuclear factor kappa-light-chain-enhancer of activated B cells (NFκB), which leads to transcription of several pro-inflammatory factors (27). Importantly, ROS has been shown to upregulate PGE2 production and PGE2 receptor expression leading to enhanced cellular proliferation (61, 62). In cancer, such as oesophageal squamous cell carcinoma, hypoxia upregulates HIF-1α, which not only leads to angiogenesis, but can cause activation the PGE2 synthesis pathway, upregulation of inflammatory IL-1β as well as activation of growth factor signalling pathways (63, 64). Just as hypoxia can lead to PGE2 synthesis, an *in vitro* study using lung cancer cells showed that PGE2 can in turn induce HIF-1α which increases VEGF (26), suggesting that a vicious cycle of hypoxia and inflammation can be triggered in certain tumours, resulting in progressive disease. Whether or not such a cycle exists in progressive OSCC disease remains to be elucidated.

### PGE2-Mediated Inflammation

The roles of PGE2, COX-1, and COX-2 in OSCC have been discussed in detail previously (3). Here, we provide additional background on PGE2 synthase enzymes and PGE2 receptors. Briefly, prostaglandins (PGs), including PGE2, are members of the eicosanoid family and are produced from arachidonic acid (AA) (65, 66) by almost all the cells of the body. PGH2 is synthesised by cyclooxygenase enzymes from AA (67), which is then converted to PGE2 by PGE synthase enzymes and exits the cell by a specific transporter named multidrug resistance protein 4 (MRP4) (68). COX enzymes include COX-1 (prostaglandin endoperoxide H synthase-1) and COX-2 (prostaglandin endoperoxide H synthase-2). They are similar in structure and catalytic activity, but they are genetically distinct and they function independently to utilise different pools of AA to synthesise prostaglandins (69–71). An important difference between these two enzymes is located in their promoter regions; the promoter region of the human COX-1 gene lacks transcriptional regulatory sequences, leading to its constitutive expression in a majority of cells (72). In contrast, COX-2 has multiple transcriptional regulatory sequences in its promoter region, and its expression can be induced by multiple cytokines (72). Reid et al. (68) found that PGE2 efflux was inhibited by nonsteroidal anti-inflammatory drugs (NSAIDs), so NSAIDs may reduce extracellular PGE2 levels by inhibiting PG synthesis and by inhibiting PGE2 secretion (68). PGE2 then acts locally through binding of one or more of its four receptors, EP1–EP4 (73).

#### Prostaglandin E_2_ Synthase Enzymes (mPGES-1, mPGES-2, and cPGES)

Microsomal prostaglandin E synthase-1 (mPGES-1) was first isolated from bovine seminal vesicles in 1977 (74). Over 20 years later, Jakobsson et al. (75) characterised human PGES and found that it had the ability to convert PGH2 to PGE2 (75). Forsberg et al. (76) studied human PGES gene structure, localisation, and regulation and concluded that mPGES-1 couples with COX-2 activity to increase PGE2 production (76). Microsomal PGES-1 is upregulated in inflammation and in cancer (77, 78). This has been demonstrated by Ramanan et al., who found that overexpressed COX-2 is linked to mPGES-1 activity and increased proliferation in different cancer cell lines including those of prostate, lung, and colon (79). Further support of the role of inflammation and PGE2 in tumour progression comes from an OSCC study showing that fibroblasts (an important stromal cell component of the tumour microenvironment) enhances PGE2 secretion and upregulates COX-2 and mPGES-1 leading to increased angiogenesis and tumour cell migration and proliferation (80).

COX-2 and mPGES-1 have been shown to be co-expressed in OSCC (81), and their expression was positively associated with proliferation and differentiation (82). In OSCC patients who had not received chemotherapy before surgery, high levels of COX-2 and mPGES-1 were associated with poor prognosis (83). To our knowledge, the impact of hypoxia on PGE2 synthase enzymes in OSCC has not been explored. However, a positive relationship between hypoxia and mPGES-1 expression was identified in a mouse model of neonatal hypoxia (84). In a mouse model of colon carcinoma, mPGES-1 expression was stimulated by HIF-2α activity, and in murine cartilage cells, mPGES-1 was stimulated by HIF-1α (85, 86). Collectively, these findings indicate that a relationship between hypoxia and mPGES-1 in OSCC is worth exploring.

The second enzyme involved in PGE2 synthesis is microsomal prostaglandin E synthase-2 (mPGES-2). Microsomal-PGES-2 is coupled with both COX-1 and COX-2 to produce PGE2 for tissue homeostasis as well as in disease and shows modest coordination with COX-2 (87). Camacho et al. (88) found that tumour samples of OSCC patients demonstrated that COX‐1 expression was correlated with mPGES‐2 expression, while COX-2 expression was not (88).

The third enzyme important in PGE2 production is cytosolic prostaglandin E synthase (cPGES). Its expression is constitutive and is coupled with COX-1 to maintain PGE2 production required for cellular homeostasis (89). Increased expression of cPGES expression has been shown in OSCC tumour samples, but it was not related to either COX-1 or COX-2 expression. The significance is unknown, but the authors noted that cPGES is identical to p23 which binds to heat shock protein 90 (a chaperone capable of protecting abnormal proteins in tumour cells from degradation), and has been implicated in carcinomas of the breast, prostate and thyroid (88).

#### Prostaglandin E2 Degradation Enzyme (15-Hydroxyprostaglandin Dehydrogenase)

15-hydroxyprostaglandin dehydrogenase (15-PGDH) is an important enzyme in the degradation pathway of the prostaglandins. Yan et al. (90) showed that 15-PGDH is highly expressed in normal intestine while its expression is inhibited in cancer of the colon, and concluded that 15-PGDH acts as a tumour suppressor (90). Tumour suppressor activity of 15-PGDH has also been shown in OSCC, including a study showing that Apricoxib (a COX-2 inhibitor) upregulated 15-PGDH in OSCC cell lines (91, 92). A relationship between hypoxia and 15-PGDH activity has not been investigated in OSCC, but in lung cancer cell lines, 15-PGDH was negatively associated with angiogenesis and COX-2 expression (93, 94). In colon cancer cell lines, hypoxia decreased the expression of 15-PGHD, which was accompanied by increases in COX-2/PGE2 (95). Future studies should be conducted to determine if hypoxia leads to increased PGE2 levels in OSCC *via* reduced expression of 15-PGHD.

#### PGE2 Receptors (EP1, EP2, EP3, and EP4)

PGE2 exerts its effects by binding to one of its four receptors, EP1–EP4 (73). The potential of EP receptors as therapeutic targets in cancer was reviewed by O’Callaghan and Houston in 2015 (96). In this section, we provide a brief overview of EP receptors and their significance to OSCC. Overall, there is evidence that all 4 receptors may be present in OSCC, but individual studies are occasionally conflicting. Physiologic functions of EP1 include thermal hyperalgesia and inflammatory nociceptive responses (97). Several studies have suggested a role for EP1 in OSCC, including a study showing that an EP1 selective antagonist (ONO-8711) reduced proliferation of OSCC in a rat model (81, 88, 98, 99). Furthermore, Yang et al. showed that overexpression of COX-2 and increased PGE2 in OSCC led to increased cellular migration and intercellular adhesion molecule 1 (ICAM) expression through EP1 but not EP2, -3 or -4, and this effect was attenuated by inhibitors of protein kinase C (98).

EP2 is expressed in OSCC (81, 100–102). Functions of EP2 outside of neoplasia include regulation of mammary gland hyperplasia induced by COX-2 (103), limitation of phagocytosis by alveolar macrophages (104), and inhibition of T cell proliferation (105). In cancer, EP2 contributes to diminished antitumor cellular immune responses *in vivo* by inhibiting dendritic cell differentiation and function (106), and has shown ability to inhibit Treg differentiation *in vitro* (107). It also regulates endothelial cell motility, survival and tumour angiogenesis *in vivo* (108).

The physiologic functions of EP3 include inhibition of inflammatory allergic reaction (109), and improved angiogenesis and lymphangiogenesis during wound healing *via* VEGF-C and VEGF-D (110). The role of EP3 in cancer is controversial, demonstrating both protective and promoting effects depending on the study. In squamous cell carcinoma of the skin, EP3 receptor deficiency either had no effect (111, 112), or was shown to contribute to squamous cell carcinoma development, but not to progression (113). On the other hand, other research groups have shown that EP3 has a pro-tumour role. For example, the EP3 antagonist (ONO-AE3-240) decreased the growth of OSCC cells *in vitro* (101), suggesting EP3 facilitates OSCC proliferation. It has been proposed that both EP2 and EP3 can increase proliferative responses to PGE2, but EP3 has been claimed to be more important than EP2 in the proliferation of OSCC (102). EP3 expression has also been shown to increase with the degree of differentiation in OSCC tumours (81, 114).

The normal roles of EP4 include participation in bone remodeling (115) and inhibition of CD4 T cell activation with subsequent downregulation of the immune response (116). PGE2 modulates macrophage function by activating the EP4 receptor and inhibiting cytokine release, while EP2 and EP4 receptors regulate antigen-presenting cell functions (105). EP2 and EP4 also have downstream signalling pathways in common, including activation of the PI3K/Akt signalling pathway, as well as activity of β-catenin (a positive mediator of tumour invasion and metastasis) (96). These are important pathways in the pathogenesis of cancer in general, and in hypoxia specifically, and will be discussed further in section 3.4. In wound healing, EP4 behaves similarly to EP3 by promoting angiogenesis and lymphangiogenesis by upregulating VEGF-C and VEGF-D expression (110), illustrating another overlapping mechanism with hypoxic response. Multiple studies have shown EP4 expression in OSCC and an EP4 antagonist (L-161,982) inhibited the proliferation in OSCC (81, 101, 117). This is in contrast with Abrahao et al. (102) who showed that all EP receptors except EP4 were expressed in OSCC (102). More recently however, Li (107) and Osawa (118) have demonstrated that EP4 does indeed function in OSCC. Li et al. showed that an EP4 inhibitor blocked PGE2-mediated signalling and proliferation in tongue OSCC cells (117). Osawa et al. showed that EP4 contributes to OSCC cellular migration and metastasis in a mouse model though a calcium selective ion channel called ORAI1 (118).

Studies evaluating the relationship between hypoxia, Prostaglandin E2 synthase enzymes and EP receptor expression in OSCC have not been published. However, hypoxia has been shown to increase PGE2 secretion and EP1 expression in a murine osteoblast cell line, but not EP2, EP3 or EP4 (62). It would be interesting to know how hypoxia impacts EP receptor expression and activity in OSCC.

### Reactive Oxygen Species and Oxidative Damage in Cancer

An important overlapping feature of hypoxia and inflammation is the generation of ROS and oxidative damage. ROS include both free radicals and non-radical derivatives of oxygen (119), such as hydroxyl radicals, hydrogen peroxide, and superoxide, and are produced inside the cell from oxygen metabolism (120, 121). ROS are capable of inducing DNA damage and genomic instability (122), leading to more inflammation,^1^ stabilisation of HIF-1^2^ and subsequent angiogenesis (123), and reprogramming of cellular metabolism (124). Interestingly, there is the potential for a positive feedback mechanism where ROS can go on to stimulate the production of PGE2 *in vitro*, which was demonstrated in a study using human keratinocytes (125). The importance of inflammation and ROS in OSCC patients is demonstrated by the association between OSCC and tobacco products (126), with tobacco use known to result in free radicals capable of DNA damage (127), an important in event in the development of OSCC (128).

The extent of oxidative damage from ROS is normally kept in check by antioxidant mechanisms that involve the activity of superoxide dismutase, glutathione peroxidase, phospholipid-hydroperoxide glutathione, and catalase (129). Pharmacologic and dietary antioxidants represent an opportunity to mitigate the harmful outcomes of ROS generated by hypoxia and inflammation in the tumour environment. This strategy will be revisited in section 4, which is dedicated to cancer therapy.

### Mechanisms Linking Inflammation and Hypoxia in Progression of OSCC

**Figure 5:**
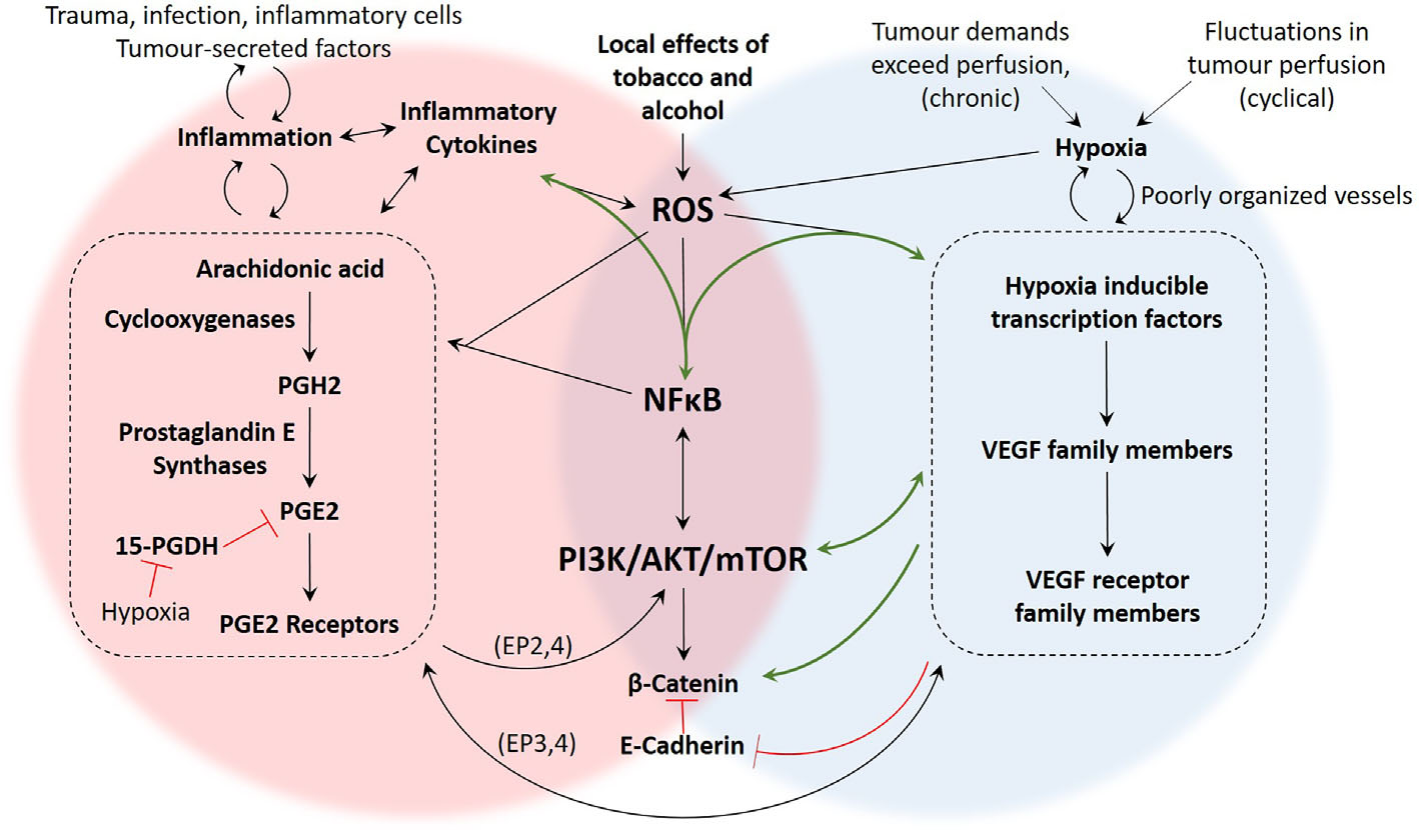
Summary of proposed mechanisms linking inflammation and hypoxia in OSCC. There are several studies demonstrating the presence and function of the molecular components of these pathways in OSCC, yet mechanistic studies investigating how these pathways intersect during hypoxia and inflammation are more limited. The green arrows represent pathways that have been demonstrated in OSCC studies such as (10, 21, 27, 28, 41). The black arrows are pathways that have been demonstrated in non-OSCC studies, including (26, 62, 110, 125, 134). The signalling pathways associated with hypoxia-driven angiogenesis and the COX2/PGE2 axis of inflammation are numerous and our understanding is constantly evolving. However, based on the literature, important areas of overlap between inflammation and hypoxia include generation of ROS, activation of NFκB, signalling through the PI3K/Akt/mTOR pathway, and activity of β-Catenin.

To this point, this review has focused on evidence for overlapping roles of hypoxia and inflammation in OSCC progression. In this section, the focus shifts to candidate mechanisms that may be responsible for this overlap. Key transcription factors include HIF-1α and NFκB, as well as a variety of cytokines and growth factors (130). NFκB is considered a sensor for oxidative stress and it regulates genes involved in inflammation, immune response, cell survival, and apoptosis (30, 131, 132), and importantly has been found to be activated in OSCC (133). NFκB can be directly activated by ROS, and therefore represents a signalling node that is common to inflammation and hypoxic pathways (**Figure 5**) (132). Additionally, NFκB can be activated indirectly *via* other inflammatory mediators such as tumour necrosis factor alpha (TNFα) and IL-1 (132). A study using a lung cancer cell line showed that inflammatory IL-1β can activate NFκB, which in turn activates the COX-2/HIF-1α/VEGF angiogenic axis (26). Although not yet demonstrated in OSCC models or patients, these findings suggest that ROS generated in the OSCC microenvironment has the potential to activate both the COX/PGE2 and HIF-1α/VEGF pathways *via* activation of NFκB.

Evidence for the importance of the NFκB and Akt signalling nodes in the COX/PGE2 pathway of inflammation and in hypoxic response was presented in previous sections. Importantly, both of these pathways are involved in OSCC invasion and metastasis. An important step in metastasis is epithelial-to-mesenchymal transformation (EMT). Kaneko et al. (135) found that hypoxia induces EMT activation in OSCC cells *via* the PI3K/Akt pathway (135). Additionally, Joseph et al. (10) recently reviewed the role of hypoxia in OSCC EMT including the contributions of NFκB and tumour associated macrophages (TAMs) (10). In general, hypoxia contributes to EMT and metastasis by reducing E-cadherin expression and increasing β-catenin availability (134). As mentioned earlier, signalling through β-catenin is one of the pathways utilised by EP2 and EP4 prostaglandin receptors (96), highlighting the possibility that hypoxia and PGE2-mediated inflammation could have converging effects on EMT through β-catenin (**Figure 5**).

In OSCC cells, reduced E-cadherin was associated with HIF expression (136), which is congruent with the finding that lymph node metastasis and tumour recurrence in OSCC patients was associated with HIF-1α and VEGF (137, 138). Similarly, the COX-2/PGE2 pathway, along with hypoxia, has been reported to contribute to OSCC metastases by increasing tumour cell migration and upregulation of intercellular adhesion molecule-1 (ICAM-1) (98, 139, 140). Several other OSCC studies have shown that hypoxia leads to increased HIF-1α and proliferation (141–143). Similarly, the COX-2/PGE2 axis promotes cell proliferation and tumour growth in OSCC (144–147). Although studies have not looked directly at the relationship between HIF transcription factors and cyclooxygenase enzymes in OSCC, both pathways appear to exert similar influences on OSCC behaviour lending support to the idea that they share common mechanisms such as NFκB activity and Akt signalling. An overview of interacting mechanisms in inflammation and hypoxia is provided in **Figure 5**.

## Role of Inflammation and Hypoxia in Cancer Treatment

### Overview of OSCC Treatment

OSCC treatment may include surgery, radiation, chemotherapy, and psychological support, with the outcome of the treatment differing for individual patients (148). As most OSCC patients are diagnosed late in the disease; they require combinations of chemotherapeutic agents with radiation (149). Chemoradiotherapy can cause complications such as dysphagia (150), cachexia, poor wound healing (151), and dryness of the mouth (152). Additionally, 30% to 50% of patients of OSCC develop malnutrition (153). In patients that are determined to be clear of OSCC after therapy, 50-60% will go on to develop local recurrence with an overall 5-year survival rate of less than 50%, and 20-30% of them developing metastasis (154).

### Impact of Hypoxia on Radiotherapy and Chemotherapy

Jing et al. (155) has recently published a review article summarizing the impact of hypoxia on cancer treatment in general (155). Here, we provide a brief overview of hypoxia in the context of treatment resistance, and then emphasize OSCC-specific studies. Radiation therapy works by causing DNA breakage (156), endothelial cell apoptosis in tumour blood vessels (157), and cell cycle arrest (156). Oxygen is essential for radiation to work as it allows for ROS production which will cause DNA damage, ultimately leading to tumour cell death (158). The tumour response to radiotherapy depends on different factors including tumour size, oxygenation, and pH (lactate levels) (159). Larger tumours require more oxygen and are more likely to become hypoxic and utilise glycolysis accompanied by acidosis (159). While DNA damage is an important feature of radiation therapy success, it has been proposed that acidosis-associated reduction in DNA repair can actually lead to accumulation of mutations allowing treatment-resistant tumour cell lineages will emerge (159–161). Tonissi et al. (162) showed that reoxygenation of OSCC cell lines before radiotherapy inhibited proliferation and sensitised the cells to radiotherapy compared to hypoxic OSCC cells. The authors concluded that hypoxia contributes to OSCC radioresistance (162).

Interestingly, one of the proposed mechanisms of radioresistance is the ability of radiation therapy to induce VEGF expression, which helps to protect tumour blood vessels from the cytotoxic effects of radiation (163). Also, it has been shown that VEGF expression after radiation contributes to the survival of cancer cells as well as tumour resistance *in vitro* (164). As mentioned above, VEGF leads to the formation of new blood vessels; however, the blood vessels are disorganised and highly permeable, leading to areas of low oxygenation and poor drug delivery (165). HIF-1 has been shown to be upregulated after radiation therapy, with the subsequent promotion of EMT and post-radiation tumour recurrence (166). The phenomenon of radiation-associated HIF-1 activity and upregulation of VEGF has led to the use of antiangiogenic drugs to help to solve the problem of radiation resistance. Counterintuitively, antiangiogenic drugs can potentially stabilise the vasculature, temporarily improving oxygenation which leads to generation of more free radicals, resulting in more DNA damage (163).

Although one of the rationales of antiangiogenic therapy is reduce tumour perfusion and therefore inhibit growth, worsening tumour hypoxia can lead to the tumour cells using anaerobic glycolysis to sustain themselves (167). Additionally, antiangiogenic therapy has been associated with tumour cells taking on a more invasive phenotype, leading to increased intravasation and metastasis (168). A clinical trial in OSCC patients showed that Sunitinib and SU5416 (a receptor tyrosine kinase inhibitor and an antiangiogenic drug) generated no treatment response, yet serious side effects such as fatigue, vomiting, and fatal haemorrhage caused the treatment to not be recommended (169, 170). Myoung et al. (171) showed that thalidomide (an antiangiogenic drug) did not inhibit OSCC as a monotherapy (171), providing support to the concept that angiogenesis inhibitors have limited effectiveness when used as a solitary agent.

Despite angiogenesis inhibitors having limited effectiveness as single-agent cancer therapy, there have been some promising results when they are combined with other medication. For example, Yoo. et al. (172) found that VEGF inhibitors combined with chemoradiation are safe and efficacious for the treatment of advanced non-metastatic OSCC (172). Hsu et al. (173) reviewed the benefits of VEGF inhibitors combined with radiotherapy in OSCC treatment, which included selective cytotoxic effects on proliferating endothelial cells (rather than non-proliferating cells), targeting of VEGF receptors in the tumour (163, 174), and less toxicity in normal tissues (175). The authors concluded that the combination of antiangiogenic drugs and radiation can improve survival as well as radiosensitivity in OSCC (173).

Under hypoxic conditions, HIF-1 reprogramming of cancer cell metabolism includes increased glucose transport into the cell and increased conversion of glucose to pyruvate (176). Targeting metabolic enzymes regulated by HIF-1 can be a therapeutic approach to cancer (176). A study focusing on OSCC revealed that HIF-1α helps cancer cells adapt to hypoxia by increasing their glucose transport and lactate production, which can contribute to OSCC radiotherapy resistance (177). Interestingly, lactate accumulation was found to be related to subsequent development of metastases in OSCC patients (178).

Based on the finding that hypoxia alters tumour metabolism, drugs capable of altering cellular metabolism have been studied as part of cancer therapy. Metformin, a biguanide, is an example of a metabolism-modifying drug, Metformin is used in the treatment of type 2 diabetes and has anticancer activities (179). It has been shown to reduce HIF-1α expression, inhibit cell proliferation and migration, and stimulate apoptosis in an OSCC cell line under hypoxic conditions (143). Combination of metformin with 5-fluorouracil inhibited *in vivo* and *in vitro* HIF-1α expression, proliferation, and invasion of OSCC. This combination was well tolerated in mice (141).

### Hypoxia and Inflammation Crosstalk in Cancer Treatment

Support for the role of hypoxia combined with inflammation in chemosensitivity is provided by Xuan and Wang (180), who showed that the inflammatory cytokine, IL-1α, was upregulated in gastric cancer during hypoxia and had a positive correlation with tumour stage, lymph node metastasis and resistance to cisplatin (180). Huang et al. (181) showed that HIF-1α stimulates COX-2 expression, invasion, metastasis, and EMT after transcatheter arterial chemoembolisation treatment in hepatocellular carcinoma patients, which contributed to poor prognosis (181). In xenograft models of melanoma and oral carcinoma, COX-2 has been shown to be upregulated in tumour endothelial cells compared to normal endothelial cells, and a COX-2 inhibitor (NS398) exhibited inhibition of angiogenesis only in tumours (182). In a nude mouse model of OSCC, the COX-2 inhibitor, celecoxib, demonstrated antiangiogenic effects characterised by inhibition of new blood vessel formation, reduced tumour volume, and chemopreventive activity if given early (183). It also decreased COX-2 and VEGF expression in OSCC as well as stimulating apoptosis and inhibiting proliferation and invasion *in vivo* and *in vitro* (184).

### Natural Products Targeting Inflammation and Angiogenesis in Cancer Treatment

Natural products have shown anti-inflammatory and anticancer activities and have gained attention as alternative and/or adjuvant therapy for conventional cancer treatments. Different types of natural products that have been investigated in OSCC include cranberries, blueberries and turmeric (curcumin).

Cranberries have been shown to contain phytochemicals with anticancer, anti-inflammatory and antioxidant activities (185–187). Cranberry polyphenols have been shown to possess antiproliferative activity in oral cancer cell lines (188), and extracts from cranberries, grapes and blueberries also inhibited proliferation of OSCC cells (189, 190). A double-blind, randomised and placebo-controlled study showed that blueberries have immunomodulatory effects, can attenuate oxidative stress and reduce inflammation in metabolic syndrome in adults. These effects were accompanied by reduced ROS levels in the blood and decreased monocyte gene expression of TNFα, IL-6, and toll-like receptor 4 compared to the placebo (191). In a hamster model of OSCC, blueberry extract inhibited angiogenesis and invasion by inhibition of NFκB activation and PI3K/Akt pathways (192). Other natural products besides cranberries and blueberries have shown anti-inflammatory and anticancer activities in OSCC cells. For example, 3-O-acetyloleanolic acid (3AOA) is an oleanolic acid derivative isolated from the seeds of *Vigna sinensis K* (cow pea), which inhibited VEGF-A production in hypoxic OSCC cells and reduced lymphangiogenesis and lymph node metastasis in OSCC *in vivo (* 193 *).* Isocudraxanthone K (a natural compound derived from Cudrania tricuspidata, a herbal remedy for inflammation and cancer), showed antiproliferative effects on OSCC cell lines by inhibition of HIF-1-α (194).

Natural products can be combined with conventional therapies in cancer treatment to decrease their toxicity and enhance their activity. For example, studies have shown that curcumin combined with chemotherapy (paclitaxel) increased apoptosis and inhibited proliferation of OSCC cells compared to chemotherapy alone (195). Curcumin also sensitised OSCC to radiotherapy *in vivo* and *in vitro*, which was accompanied by decreased COX-2 expression (196).

Despite studies showing the success of natural products in cancer treatment, there is still controversy about their safety and bioavailability and most of these products are not approved as a therapeutic agents. Wang et al. (197) reviewed herbal products for their bioavailability, side effects and interactions with prescription drugs, leading them to conclude that they are not suitable for all types of patients and should be taken cautiously (197). In summary, the ability of natural products to inhibit hypoxic and inflammatory mechanisms in OSCC treatment needs more investigation.

## Comparative Oncology: Inflammation and Hypoxia in Domestic Animal Cancer

### Comparative Biology of Tumour Hypoxia

Although studies of angiogenesis in animal cancer are relatively limited compared to human cancer research, HIFs and VEGF have been shown to have critical roles during placentation in cats (198). In the context of cancer, Brown et al. (199) showed that HIF-1α upregulation is responsible for increased VEGF in feline OSCC cell lines (199). Harris et al. (200) showed that VEGF-D, VEGF-A and HIF-1α are also expressed in FOSCC tissues and cell lines, and they confirmed that FOSCC cells grown in hypoxic conditions express increased levels of VEGF-D. They also determined that HIF-1α expression was related to Twist expression (a marker for EMT), which they proposed could serve as a therapeutic target (200). These findings demonstrate that, like their human counterparts, feline OSCC cells are capable of hypoxia-induced expression of VEGF, and HIF-1α appears to play a role in EMT.

Other studies have demonstrated VEGF expression in animal cancer. Millanta et al. (201) found that VEGF expression is a prognostic indicator in invasive feline mammary carcinomas, as unfavourable prognosis was associated with a higher percentage of VEGF-positive cells (201). An angiogenesis drug (bevacizumab) suppressed tumour growth in a xenograft model of VEGF-expressing feline mammary carcinoma (202). VEGF can have a prognostic value in the early detection of oral neoplasms (squamous cell carcinoma, fibrosarcoma, and melanoma) in dogs, as it is negatively associated with prognosis (203). Further support of the role of inflammation in angiogenesis in comparative oncology is demonstrated by a study showing that COX-2 expression was significantly correlated with VEGF expression in feline and canine cutaneous SCCs and OSCC tissues (204).

Our previous work found that a combination of a bisphosphonate bone resorption inhibitor (zoledronic acid) and an NSAID (meloxicam) was well tolerated and reduced tumour growth in an orthotopic mouse model of bone-invasive FOSCC (205). Interestingly, another group of researchers showed that zoledronic acid caused a reduction in circulating serum VEGF in feline OSCC patients and inhibited tumour-induced angiogenesis *in vivo* and *in vitro* (206). The authors of that study speculated that zoledronic acid may have reduced VEGF levels by reducing the release of bone‐derived TGF‐β (an inducer of VEGF expression), or by reducing VEGF release by OSCC cells due to inhibited secretion, or due to death of VEGF-secreting OSCC cells.

Feline immunodeficiency virus (FIV) is related to the development of feline cancer (207). Biezus et al. (208) found that FIV can cause an imbalance of oxidant-antioxidant mechanisms with increased ROS and DNA abnormalities, supporting of the role of inflammation, oxidative stress and cancer development in cats (208). Allemann et al. (209) found that there are heterogonous areas of hypoxia in feline fibrosarcomas (209), and HIF-1α expression is has been related to angiogenesis and poor prognosis in canine mammary cancer (210). VEGF and HIF-1α have been shown to be upregulated in feline OSCC cell lines and tissues, with STAT3 activation leading to increased proliferation and reduced apoptosis in OSCC cell lines (199). Similarly, Cannon et al. (211) found that the downregulation of protein kinase CK2 [an activator of NFκB (212) and PI3K/Akt (213)] in feline OSCC can cause increased OSCC apoptosis (211). These limited studies support the role of hypoxia, ROS, and inflammation in tumours of cats and other animals, but the underlying mechanisms require further investigation.

### Comparative Biology of PGE2 Synthesis and Signalling in Cancer

It has been previously shown that feline OSCC cell lines (SCCF1, SCCF2, and SCCF3) express COX-1 and COX-2 and secrete PGE2 in a cell line-specific manner (7). Since cats have demonstrated responsiveness to cyclooxygenase inhibitors (214), it would be expected that PGE2 synthase enzymes and EP receptors would be present in feline tissues and cells. To date, these receptors and synthase enzymes have not been published in feline OSCC. In contrast, Van den Top et al. (215), found that mPGES-1 was expressed in papilloma and SCC in horses, and the expression was strongest in well-differentiated tissue compared with poorly differentiated tissue (215). Millanta et al. (216), found that mPGES-1 and EP2 receptor was expressed in canine osteosarcoma, as opposed to their lack of expression in normal canine bone (216). In another canine study, EP1, EP2, EP3, and EP4 were present in urinary bladder and urethra, with expression varying with the region and tissue layer of the lower urinary tract. There was a higher expression in the proximal urethra compared to other regions (217). Additionally, mPGES-1 and EP2 receptor expression was found to be significantly higher in canine and feline mammary carcinomas compared to adenomas and non-neoplastic mammary tissues (218).

### Impact of Inflammation and Hypoxia on Treatment of Animal OSCC

Traditional NSAIDS and COX-2 selective inhibitors are widely used in animals. In cats, they are used for preoperative pain and osteoarthritis (219), but they can cause increased motility of the small intestine, intestinal damage (220), as well as gastroduodenal perforation (221). Even though there are side effects for NSAIDS, they are still used in cancer treatment due to their anticancer and pain-relieving properties. For example, celecoxib and citrate showed antitumor activity (apoptotic and antiproliferative effects) in a canine mammary tumour cell line, individually and in combination, overcoming chemoresistance (222). Mavacoxib is a relatively new selective COX-2 inhibitor used for the management of inflammatory disease in dogs, with improved tolerance, and has been shown to possess antiproliferative and apoptotic effects in a canine cancer cell lines representing osteosarcoma, glioma, lymphoma, mast cell tumour, and hemangiosarcoma (223). In recent years, a selective prostaglandin receptor (EP4) antagonist, Grapiprant, has been approved for the management of pain associated with degenerative joint disease in dogs, and has been determined to be well tolerated in research cats (224). To our knowledge, there have been no published studies evaluating the antineoplastic potential of Grapiprant in canine or feline tumour cells.

Piroxicam can cause a decrease in tumour size with increased apoptosis in invasive transitional cell carcinoma of the urinary bladder in dogs (225). Moreover, it has shown to have anticancer activity at a well-tolerated dose of 0.3 mg/kg daily in cats with OSCC, causing more than 50% reduction of the tumour volume in two cats (there was no effect of tumour progression in nine cats with OSCC) (226). Additionally, NSAIDs (piroxicam and indomethacin) reduced the development of OSCC in rats with precancerous lesions of the tongue to 23%–31% compared to 71% in rats that did not receive NSAIDs (227).

NSAIDs can be used in cancer treatment as an adjuvant to augment the effectiveness of conventional therapy in animals. For example, firocoxib in combination with radiotherapy is safe and improved life quality (activity and appetite) in dogs with nasal carcinomas (228). Furthermore, a combination of a receptor tyrosine kinase inhibitor (Masitinib, AB1010) and NSAIDs (piroxicam) inhibited the proliferation of OSCC feline and canine cell lines as compared to either drug alone (229). Similarly, meloxicam (a COX-2 selective inhibitor) reduced feline OSCC xenograft growth in nude mice and was well tolerated when combined with a bisphosphonate bone resorption inhibitor (zoledronic acid) (205).

Despite promising studies, the use of NSAIDs as part of cancer therapy in cats is controversial. A retrospective study of 73 cats receiving long-term daily piroxicam ranging from 1 to 38 months, indicated that it is well tolerated in cats at conventional doses (230). A retrospective study (2009-2013) showed that a combination of NSAIDs with toceranib phosphate (Palladia) was well tolerated in feline OSCC. Unfortunately, the benefit of adding NSAIDS could not be determined, because only three cats (of 32) were still alive at the end of the study due to tumour progression (231). While another retrospective study of 23 cases with mammary gland adenocarcinoma treated with chemotherapy, surgery and a COX-2 inhibitor (meloxicam) showed that there was no impact on the prognosis or the survival (232, 233).

As mentioned earlier, the PI3K/Akt signalling pathway may serve as a link between inflammatory and hypoxic pathways in human OSCC. Interestingly, combination of chemotherapy (doxorubicin), its derivative (AD198) and an inhibitor of the PI3K/Akt pathway was anti-proliferative and pro-apoptotic in canine and feline OSCC cells (234). Also, the combination of etanidazole (hypoxic cell sensitiser) and radiation caused a 70% regression of tumour volume in cats with OSCC. Although it was well tolerated, unfortunately it was not curative, with all cats succumbing to tumour complications by the end of the study (235).

As mentioned above, natural products have shown promise in human cancer studies for their anti-inflammatory and antioxidant abilities and investigators have started to evaluate them as cancer therapy for animals. Most studies have been in canine patients, with cats and other species appearing less frequently in the literature. Natural products such as blueberries, turmeric, grapes, and curcumin have been tested in dogs. For example, sled dogs fed blueberries while exercising showed a significant increase in blood antioxidants compared to dogs fed a control diet (236). Martineau et al. (237) showed that polyphenol-rich extracts of blueberries and grapes were safe to be used in dog diets without affecting their renal or hepatic functions (237). Turmeric and rosemary were found to be pro-apoptotic in a variety of canine cancer cell lines (mastocytoma, mammary carcinoma, osteosarcoma) (238). Furthermore, adding turmeric and rosemary to chemotherapy augmented the antiproliferative response of cancer cells lines (239). Leray et al. (240) showed that curcumin in obese cats reduced serum acute-phase protein as well as IL-2 mRNA expression in peripheral blood mononuclear cells, leading the authors to conclude that curcumin has anti-inflammatory activity in cats and a protective effect on the feline liver (240). To our knowledge, studies of blueberries and blueberry derivatives in cats have not been published.

## Conclusion

Research has revealed that hypoxia and PGE2-mediated inflammation in the tumour microenvironment contribute to tumour progression and treatment resistance in OSCC as well as in other forms of cancer. Continued investigation of the interacting mechanisms between hypoxia and inflammation will help improve current therapies, not only for people, but for animals with OSCC. Although much is known about the interacting roles of hypoxia and inflammation, controversies still exist regarding the contributions of specific elements such as EP3 receptor and HIF isoforms. There is evidence, both for and against, the use of drugs capable of targeting inflammation and angiogenesis as part of cancer therapy, necessitating continued study. Ideally, therapies will be developed that interrupt the cycle of inflammation and hypoxia in OSCC tumours, leading to slower progression and improved response to therapy. The fact that pro-neoplastic effects of hypoxia- and inflammation-related oxidative damage also participate in the desired effects of radiotherapy, means that targeting these pathways must be carefully investigated in the context of adjuvant therapy. Although there has been much focus on inhibition of cyclooxygenase enzymes, specific inhibition of PGE2 synthase enzymes and receptors deserve more study. Similar challenges are revealed in research related to domestic animals. Continued research in the field of veterinary oncology will help animal patients survive longer and with greater quality of life. Additionally, the relatively short life span of pet animals compared to humans, combined with their intact immune systems and diverse genetics, make them valuable resources for the study of novel treatment strategies that could improve outcomes for people.

## Author Contributions

Writing-Original Draft Preparation, WN. Writing-Review and Editing, CM. All authors contributed to the article and approved the submitted version.

## Funding

We acknowledge the support of the Natural Sciences and Engineering Research Council of Canada (NSERC), [RGPIN-2019-06898, CM] and the Mitacs Training Award [WN].

## Conflict of Interest

The authors declare that the research was conducted in the absence of any commercial or financial relationships that could be construed as a potential conflict of interest.
